# Solid-State Ion-Conducting Multiblock Terpolymers

**DOI:** 10.1021/acs.macromol.5c02391

**Published:** 2025-12-23

**Authors:** Rui Sun, Yossef A. Elabd

**Affiliations:** Artie Mcferrin Department of Chemical Engineering, 14736 Texas A&M University, College Station, Texas 77843, United States

## Abstract

Solid-state multiblock copolymers with two distinct chemistries are well explored and reveal that three-dimensional (3D) continuous network morphologies result in the highest ion conductivities, morphology factors (normalized ion conductivity), and subsequently the highest electrochemical performance. However, these polymers exhibit a limited set of 3D morphologies over a narrow compositional range. Solid-state multiblock terpolymers with three distinct chemistries significantly enhance the accessible phase space and yield more 3D continuous network morphologies over a broader compositional range, yet they are relatively unexplored. In this perspective, solid-state ion-conducting multiblock terpolymers are reviewed, and the results highlight more observed 3D continuous network morphologies and exceptionally higher morphology factors compared to their multiblock copolymer counterparts. To date, only 12 morphologies have been observed in ion-conducting multiblock terpolymers. Future investigations with targeted synthesis can discover many more morphologies, and therefore unlock a new set of materials with ultrahigh ion conductivities and electrochemical performance.

## Graphical abstract

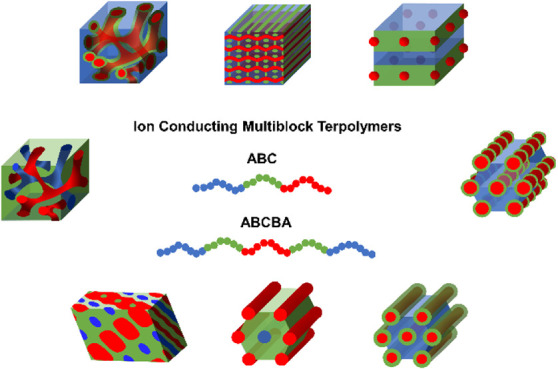

## 1 Introduction

Ion-conducting block polymers are a distinct class of materials that consist of an ion-conducting block covalently and sequentially bonded to one or more nonionic (or neutral) blocks and can form precisely controlled periodic microdomains without macroscopic separation.^1,2^ In the solid state, these materials have demonstrated promising performance in a variety of electrochemical applications, including energy conversion and storage devices,^3–6^ actuators,^7,8^ and sensors,^9^ due to their ability to incorporate multiple desired properties (e.g., high ion conductivity, high mechanical strength, high modulus) in a single versatile material platform. Specifically, there is a direct relationship between ion conductivity and electrochemical performance, and numerous studies have demonstrated that the formation of well-defined nanostructures with continuous ionic domains significantly increases ion conductivity and subsequently electrochemical performance.^3–6,10–12^

Figure 1 presents three examples of ion conductivity in microphase-separated ion-conducting diblock copolymers. Elabd, Winey, and coworkers^13^ reported an order of magnitude higher conductivity in an ion-conducting diblock copolymer with lamellar morphology compared to its analogous random copolymer with no microphase separation (Figure 1a). The conductivity was also higher than that of the pure ionic homopolymer, despite lower conducting phase volume fractions in the diblock copolymer. Liberatore, Herring, Knauss, and coworkers^14^ also observed an order of magnitude higher conductivity in an ion-conducting diblock copolymer compared to homopolymer blends with similar conducting phase volume fractions (Figure 1b). The conductivity difference in both studies was attributed to the confinement of ions in microphase-separated domains, which increases localized ion concentration and accelerates ion conduction. Additionally, the impact of morphology on ion conductivity can be quantified following earlier work by Sax and Ottino^15^ on the transport of small molecules in phase-separated polymers. A morphology factor (*f*, normalized ion conductivity) can be calculated by

$$f=\frac{\sigma }{\sigma_{c}\phi_{c}}$$

where σ is the measured ionic conductivity of the ion-conducting block polymers, σ_c_ is the intrinsic ion conductivity of the conducting phase, and ϕ_c_ is the volume fraction of the conducting block. An ideal morphology factor (i.e., for polymers without any grain boundaries, tortuous paths, or dead ends) differs between morphology types based on their dimensional connectivity. Specifically, the ideal morphology factor is 1/3 for hexagonally packed one-dimensional (1D) cylinders, 2/3 for two-dimensional (2D) lamellae, and 1 for three-dimensional (3D) gyroid networks (Figure 1c).^15–17^ For instance, Winey and coworkers^18^ reported a higher morphology factor (*f*) in an ion-conducting diblock copolymer exhibiting 3D network morphology compared to those with 2D lamellar or 1D cylindrical morphologies (Figure 1c), suggesting that ion transport is highly dependent on morphology types. The 3D network morphology contains continuous ionic domains with reduced grain boundaries or dead ends, which promotes the highest ion conductivity by facilitating the movement of ions along long-range, well-connected pathways.

**Figure 1:**
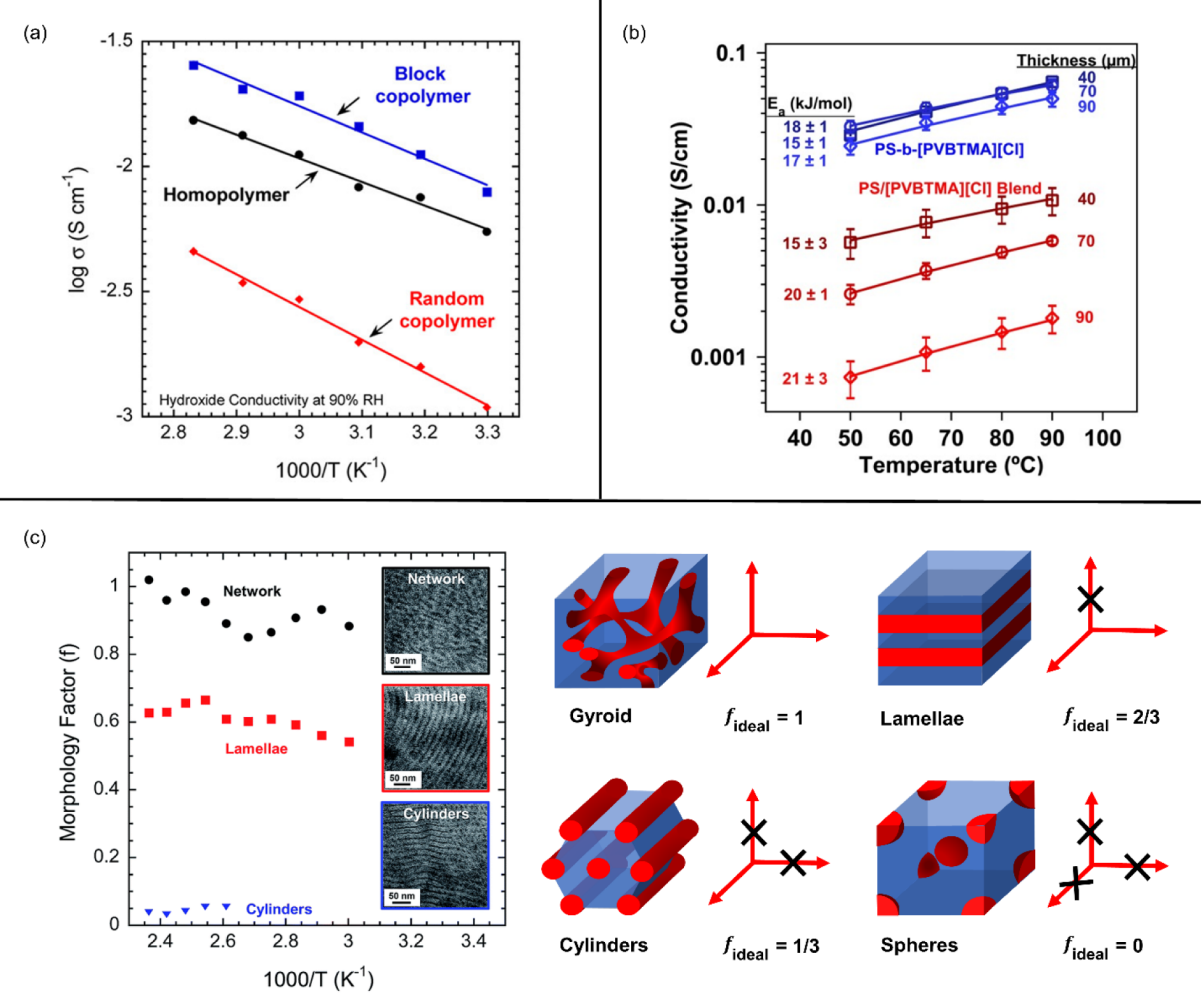
(a) Temperature-dependent ion conductivity of ion-conducting diblock copolymers and their analogous random copolymer and homopolymer. Adapted with permission from ref 13 (Copyright 2013 American Chemical Society). (b) Temperature-dependent ion conductivity of ion-conducting diblock copolymers and their analogous polymer blends. Reprinted with permission from ref 14 (Copyright 2014 Wiley Periodicals, Inc.). (c) Morphology factor (*f*) of an ion-conducting diblock copolymer with different morphology types, adapted with permission from ref 6 (Copyright 2015 The Royal Society of Chemistry), and illustrations of ideal morphology factor (*f*_ideal_) for different morphology types.

However, to date, the majority of experimental and theoretical studies focus on ion-conducting block copolymers with two chemistries (e.g., AB diblock copolymers,^3–6,10–14,18–22^ ABA triblock copolymers,^7,8,10,23^ and multiblock AB copolymers^24–31^ ). These polymers exhibit a limited set of morphologies, including conventional lamellar, cylindrical, and spherical morphologies, and scarce examples of additional morphologies, including double gyroid, O^70^ (*Fddd*), Frank–Kasper σ-phase, and Frank–Kasper A15 morphologies over a narrow composition range,^32–34^ limiting the capability to achieve well-defined 3D continuous morphologies for faster ion conduction.^35^

Studies on neutral multiblock terpolymers reveal that the introduction of a third distinct chemistry substantially enriches the phase behavior and results in more 3D continuous morphologies compared to diblock copolymers (Figure 2).^35^ Compared to AB diblock copolymers, the equilibrium morphology of ABC multiblock terpolymers is governed by more molecular variables or degrees of freedom, including the total degree of polymerization (*N*), three Flory–Huggins segmental interaction parameters (χ_AB_, χ_AC_, and χ_BC_), two independent block compositions (ϕ_A_ and ϕ_B_), and three different block sequences (ABC vs ACB vs CAB).^32^ Chang and Bates^36^ reviewed the self-assembly of ABC triblock terpolymers and highlighted the concepts of frustration and interfacial tension, which were first proposed by Zheng and Wang^37^ and later simplified by Bailey et al.^38^ ABC triblock terpolymers were categorized into three groups based on the magnitude of the interaction parameter χ_AC_ relative to χ_AB_ and χ_BC_ (Figure 3). When A/C interfaces are less miscible than the connected A/B and B/C interfaces (χ_AC_ ≥ χ_BC_ ≥ χ_AB_, Figure 3a), the end blocks strongly phase separated from each other, creating an unfrustrated scenario where conventional morphology types and their three-phase analogues similar to AB diblock copolymers were observed.^39–43^ In contrast, when A/C interfaces are more miscible than the connected A/B and/or B/C interfaces (χ_BC_ ≥ χ_AC_ ≥ χ_AB_ or χ_BC_ ≥ χ_AB_ ≥ χ_AC_, Figure 3b and c), complex equilibrium morphology might be induced due to the frustration caused by the competing effects between the chain stretching and the more favorable A/C interfaces.^36,44^ A vast array of exotic morphology types, such as three-phase continuous morphologies,^42–44^ three-phase core–shell morphologies,^45^ the knitting pattern,^46^ and hierarchical X-in-Y or X-on-Y structures (X, Y represent sphere, cylinder, or lamellae)^47–50^ has been observed in frustrated ABC triblock terpolymers (selected examples shown in Figure 2a).^35,36^ Furthermore, Shi and coworkers^51^ performed a theoretical study on the phase behavior of linear ABCBA pentablock terpolymers and their analogous ABC triblock terpolymers via 3D self-consistent field theory. Phase diagrams (Figure 2b) showed that ABCBA pentablock terpolymers achieve a more diverse set of 3D continuous networks with larger composition windows compared to ABC triblock terpolymers, while the packing frustration was smaller due to the symmetric chain architecture with the same end-block chemistry, resulting in less exotic morphology types for the pentablock terpolymers. Experimental^52–54^ and theoretical studies^51^ on the self-assembly of neutral ABCBA pentablock terpolymers have been summarized in previous reviews.^55^

**Figure 2:**
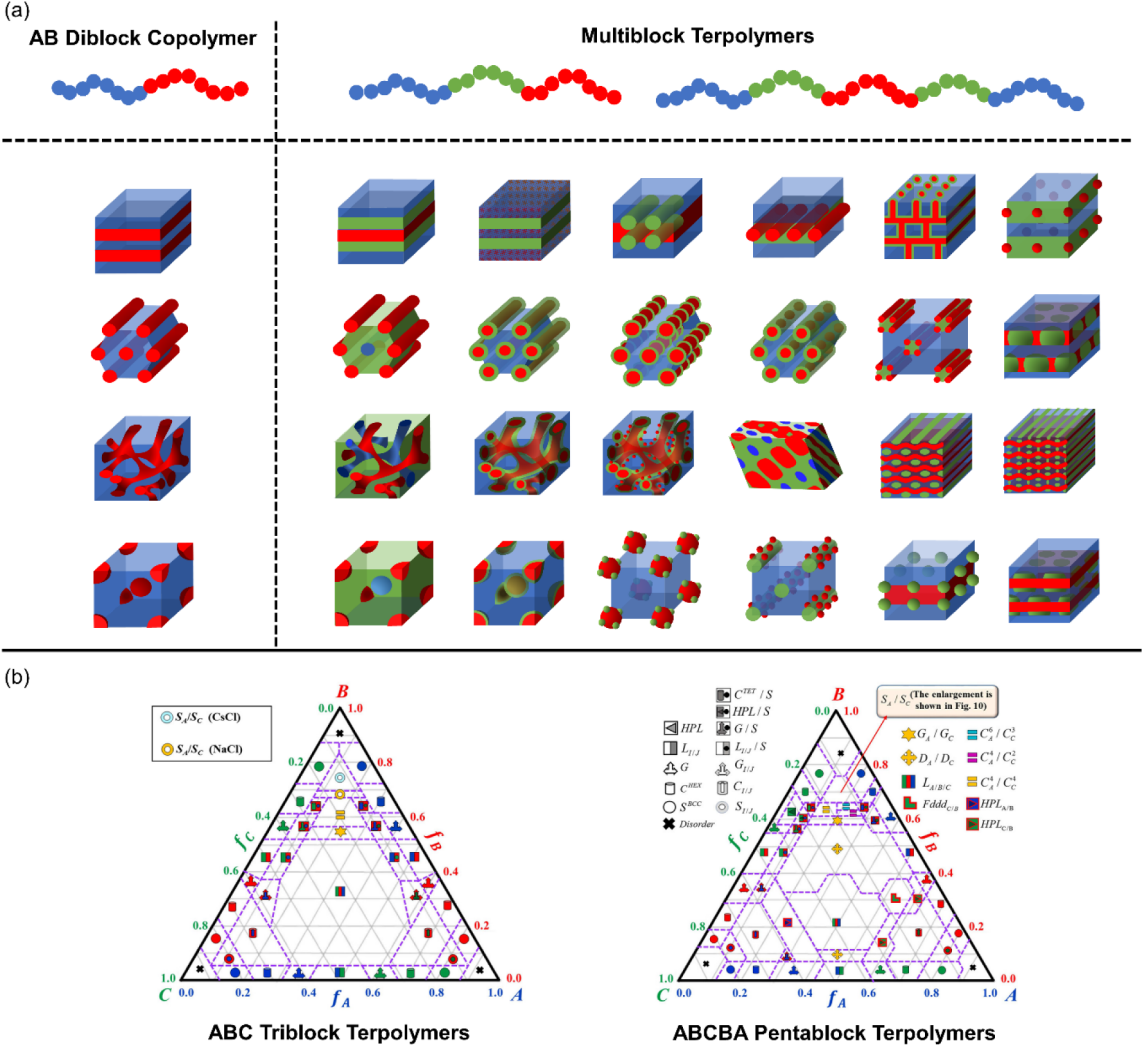
(a) Illustrations of morphologies observed or predicted in ion-conducting diblock copolymers with two chemistries and multiblock terpolymers with three chemistries. (b) Theoretical phase diagrams of ABCBA linear pentablock terpolymers and ABC linear triblock terpolymers using 3D self-consistent field theory. Adapted with permission from ref 51 (Copyright 2015 American Chemical Society).

**Figure 3:**
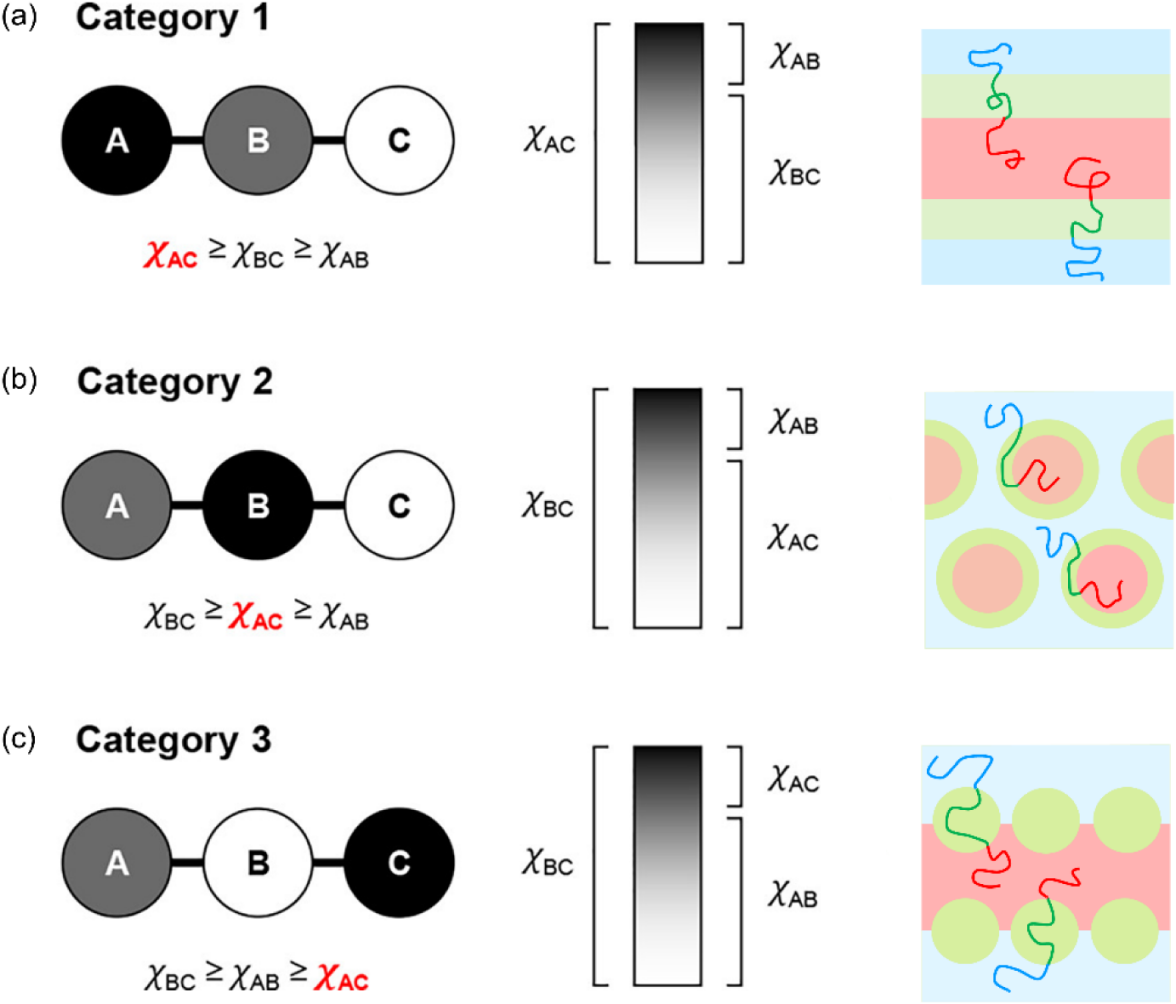
Categories of ABC triblock terpolymers based on the contrast of χ between the blocks and example illustrations of polymer chain packing: (a) χ_AC_ ≥ χ_BC_ ≥ χ_AB_, (b) χ_BC_ ≥ χ_AC_ ≥ χ_AB_, (c) χ_BC_ ≥ χ_AB_ ≥ χ_AC_. Adapted with permission from ref 36 (Copyright 2020 American Chemical Society) and ref 56 (Copyright 1999 American Institute of Physics).

Although there are numerous investigations on the phase behavior of neutral multiblock terpolymers, the morphology of solid-state ion-conducting multiblock terpolymers is relatively unexplored. Charges and electrostatics introduce additional parameters, such as ion type, concentration, and distribution, into polymer design, leading to unconventional phase behavior and unique morphologies.^10,57,58^ Therefore, a significant question remains: How does the incorporation of charge affect the morphology and, subsequently, the ion conduction of multiblock terpolymers with three distinct chemistries? Herein, we review the limited studies on ion-conducting multiblock terpolymers with three distinct chemistries and highlight the phase behavior and their impact on ion transport. Similarities and differences in ion-conducting multiblock terpolymers with regards to the impact of chain architecture (ABC vs ABCBA) and the number of mobile ions (single-ion conducting vs multi-ion conducting) are described. In addition, with a multitude of unexplored morphologies among ion-conducting multiblock terpolymers with three chemistries, future research directions are proposed to explore materials with high ion conductivities and, subsequently, potentially higher electrochemical performance.

## 2 Ion-Conducting Multiblock Polymers

This section provides an overview of the investigations on the morphology and ion conductivity of solid-state ion-conducting multiblock terpolymers with three chemistries. The studies are categorized into four groups based on the chain architecture and number of mobile ions in the polymers: (1) single-ion conducting ABCBA pentablock terpolymers, (2) multi-ion conducting ABCBA pentablock terpolymers, (3) multi-ion conducting ABC triblock terpolymers, and (4) single-ion conducting ABC triblock terpolymers. Example chemical structures of each group of polymers are shown in Figure 4, and observed morphologies are listed in Table 1. Specifically, single-ion conducting polymers refer to polymers containing one type of mobile ion with the counterion covalently bound to the polymer, while multi-ion conducting polymers refer to polymers doped with salts or ionic liquids (ILs), where two or more types of ions, including both cations and anions, are mobile. The number of mobile ions has been shown to significantly influence the morphology, overall ionic conductivity, ion transport mechanisms, and electrochemical performance of ion-conducting diblock copolymers,^3,16^ yet there are limited studies on multiblock terpolymers. Therefore, it would be of interest to discuss the observed differences in these properties between single-ion conducting and multi-ion conducting multiblock terpolymers. In addition, numerous studies have revealed that ion transport is highly dependent on the conducting ion chemistry (hydrophilic vs hydrophobic) and the hydration state of the polymers. Under anhydrous states, ion transport is strongly coupled with polymer chain segmental motions and follows Vogel–Fulcher–Tammann (VFT) behavior as a function of temperature, while ion transport under hydrated states is governed by the ion-hopping mechanism and exhibits Arrhenius behavior.^6^ For each category of ion-conducting multiblock terpolymers, the ion transport mechanisms were discussed in relation to their ion type and hydration state.

**Figure 4:**
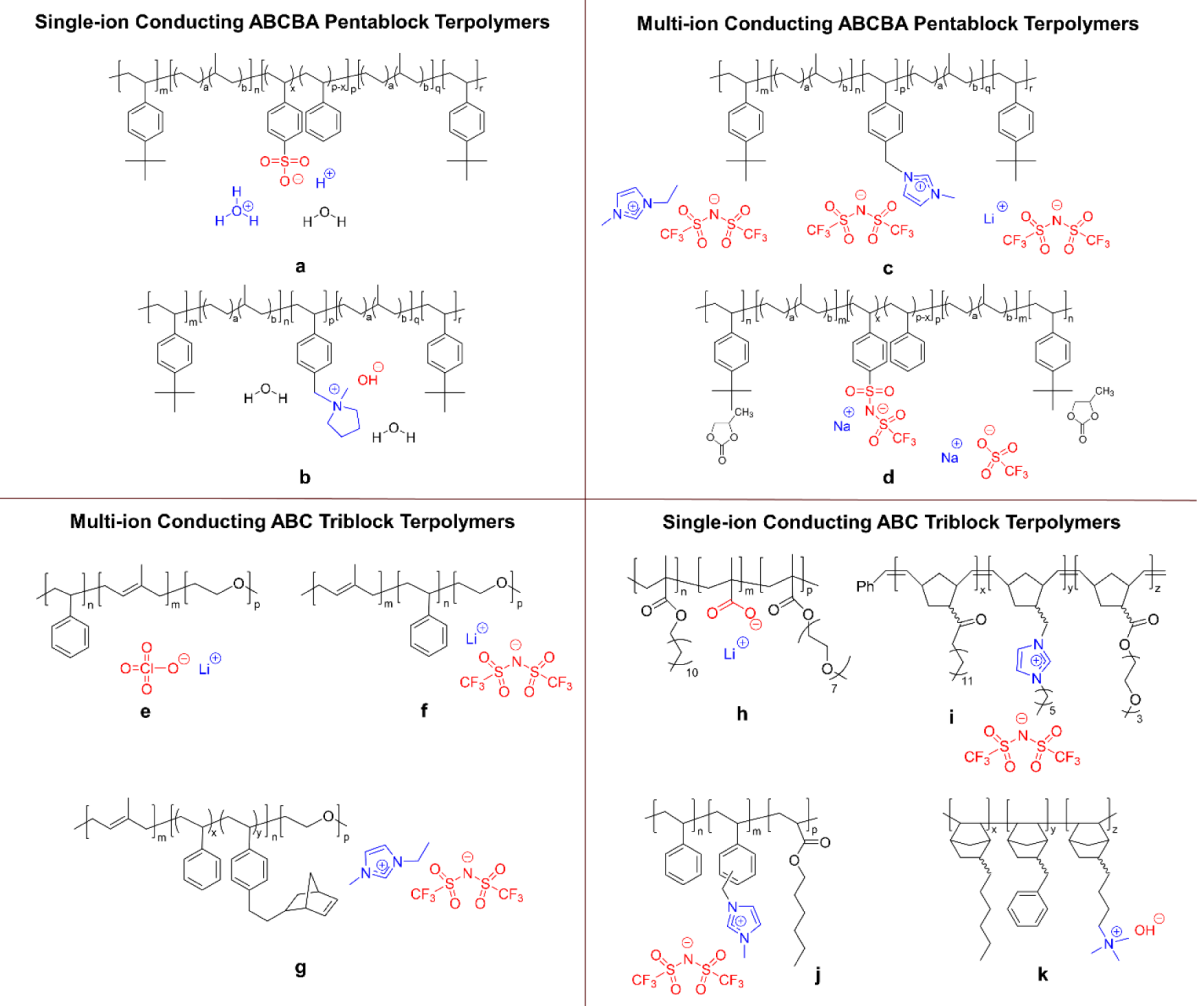
Chemical structures of selected single-ion conducting and multi-ion conducting multiblock terpolymers with three chemistries.^35,59–68^

**Table 1:**
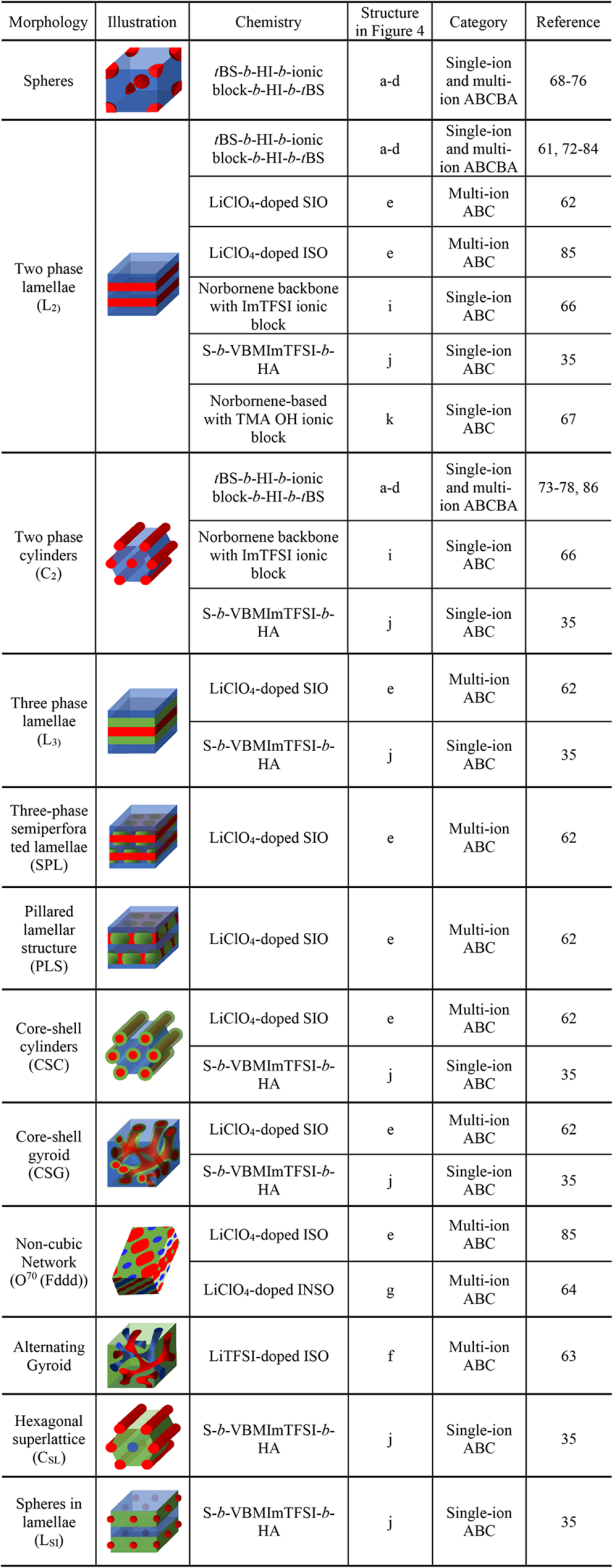
Morphology in Ion-Conducting Multiblock Terpolymers

### 2.1 Single-Ion Conducting ABCBA Pentablock Terpolymers

The first single-ion conducting ABCBA pentablock terpolymer, poly­(*tert*-butylstyrene-*b*-hydrogenated isoprene-*b*-sulfonated styrene-*b*-hydrogenated isoprene-*b*-*tert*-butylstyrene) (*t*BS-*b*-HI-*b*-SS-*b*-HI-*b*-*t*BS), was developed by Kraton Polymers LLC (NEXAR) (chemical structure: Figure 4a).^87,88^ This symmetric ABCBA pentablock terpolymer contains outer *t*BS blocks for mechanical strength, inner HI blocks for film toughness and flexibility, and a middle SS block for ion and water transport. Compared to other sulfonated block copolymers, the ABCBA pentablock terpolymer conjoins the attributes of all three chemistries and exhibits high proton conductivity, high water permeability, and enhanced mechanical stability under hydration. NEXAR is considered a promising hydrocarbon-based alternative to the benchmark perfluorinated Nafion in various applications over the past two decades, including nanocomposites,^89,90^ stimuli-responsive materials,^91^ water desalination and purification,^92–97^ flow batteries,^73^ fuel cells,^59,73,98^ gas separations,^99^ dehumidification,^100,101^ photovoltaics,^78,102^ and actuators.^103^

Early investigations of proton-conducting ABCBA pentablock terpolymers focused on their morphology in both solution^104–108^ and solid state,^69,71,72,75,76,108–111^ and their subsequent impact on transport properties. While the neutral pentablock terpolymers attained periodic nanostructures, sulfonated ABCBA pentablock terpolymers have a higher probability of being kinetically trapped during solvent evaporation and achieving nonequilibrium morphology due to their complex macromolecular structures, strong interblock incompatibility, and ion aggregation stemming from the electrostatic interactions among sulfonate groups.^69,76^ When cast from nonpolar solvents, microphase separation without long-range order was observed at all sulfonation levels, attributed to extended polymer chain conformations as a result of significantly increased χ parameters between ionic and nonionic blocks.^69,71^ This affords a unique opportunity to tune the morphology of solid-state NEXAR by changing ion exchange capacities (IECs) (i.e., degree of sulfonation)^69,71,108^ or film casting and processing conditions. Increasing the IEC from 1.0 to 2.0 m_eq_/g results in a morphology transition from discrete to interconnected SS microdomains, leading to a 10-fold increase in water vapor transport rate (WVTR)^69^ and a 50-fold increase in proton conductivity (Figure 5a).^71^ This drastic enhancement in proton and water transport is attributed to the synergistic effect of the formation of continuous ion/water transport pathways and the plasticization of the ionic domains with higher water content, i.e., a water-assisted ion transport mechanism similar to that of Nafion and other sulfonated block copolymers.^4^ On the other hand, solvent polarity and processing methods can alter the conformation, entanglements, and ion clustering of polymer chains, and significantly influence nanostructure formation via different solvent–ionomer interactions.^72,76,109,110^ Switching from nonpolar to polar solvents caused the ionic ABCBA pentablock terpolymer chains to expand and reorganize. Multiple studies from Cornelius and coworkers^73,74,112,113^ and Spontak and coworkers^75^ observed a morphology change from randomly dispersed spherical SS domains (cast from nonpolar solvent) to a well-connected SS network containing embedded nonionic lamellae or hexagonally packed cylinders (cast from polar solvent) (Figure 5b). The morphology change led to a 10-fold increase in proton conductivity at a constant IEC, and significantly enhanced performances in fuel cells, vanadium redox-flow batteries, and actuators.^73,74^ Stein and coworkers^80^ also investigated the morphology and ion transport of NEXAR as a function of polar solvent composition (wt % n-propanol in toluene). A significant increase in proton conductivity normalized by water content was observed above 40 wt % n-propanol, which is associated with a morphology transition from ordered lamellae to a disordered SS network (Figure 5c). The impact of the water content was decoupled by the normalization, further indicating that continuous ion-conducting domains promote faster ion transport.

**Figure 5:**
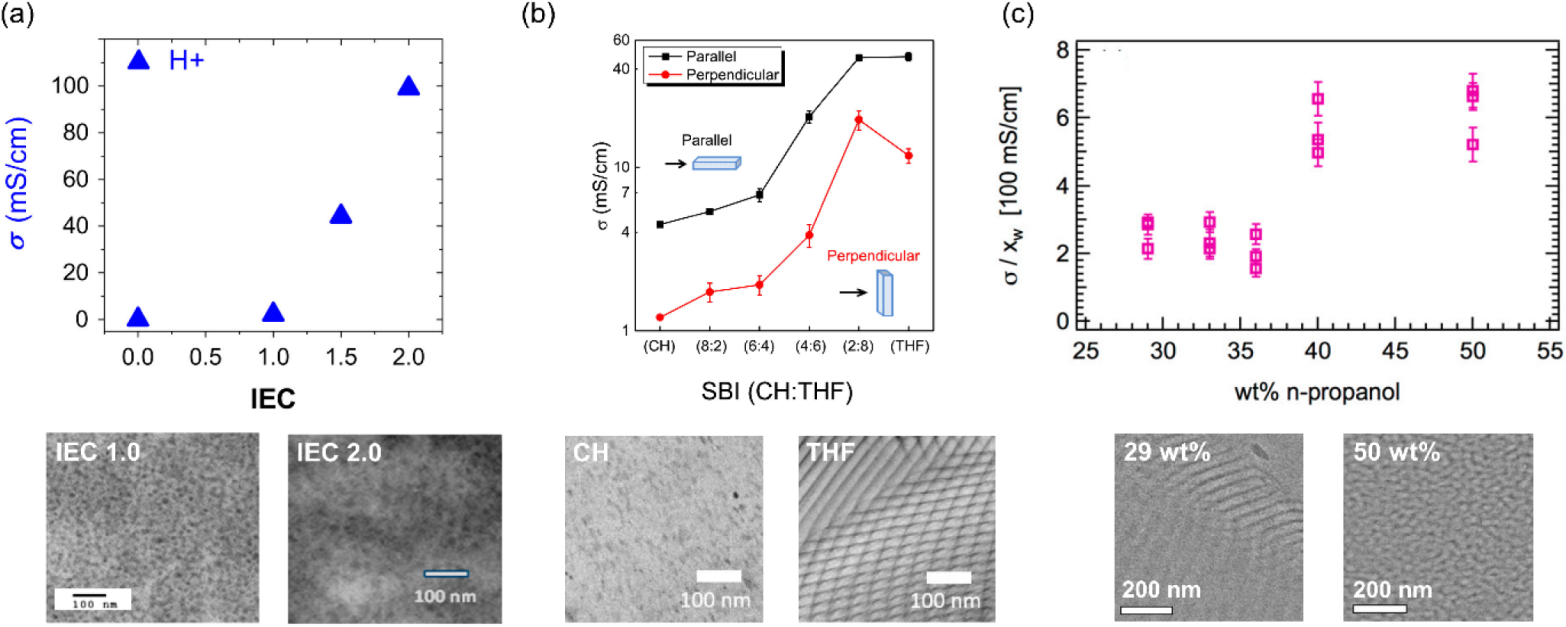
Examples of ion conductivity and TEM images of single-ion conducting ABCBA pentablock terpolymers as a function of (a) IEC, adapted with permission from ref 71 (Copyright 2014 Elsevier B.V.), (b) solvent composition (CH:THF), adapted with permission from ref 74 (Copyright 2016 Elsevier Ltd.), and (c) solvent composition (wt % n-propanol in toluene), adapted with permission from ref 80 (Copyright 2019 American Chemical Society).

Hydroxide- and bromide-conducting ABCBA pentablock terpolymers consisting of the same polymer backbone with an alkaline-stable C block (chemical structure: Figure 4b) were investigated as anion exchange membranes (AEMs) for alkaline fuel cells.^60,79,86,114^ Despite differences in middle block chemistry, hydroxide-conducting ABCBA pentablock terpolymers also exhibit weakly ordered microphase separation, which is anticipated due to their similarity to proton-conducting ABCBA pentablock terpolymers in molecular variables, i.e., similar block volume fractions with only minimal differences in ionic group sizes, the same total degree of polymerization, and comparable χ values between ionic and nonionic blocks. Ion conductivity also increased with improved ionic domain connectivity and follows a water-assisted transport mechanism similar to the proton-conducting ABCBA pentablock terpolymers.

### 2.2 Multi-Ion Conducting ABCBA Pentablock Terpolymers

Incorporating ionic small molecules (e.g., ILs and salts) into single-ion conducting pentablock terpolymers results in multi-ion conducting ABCBA pentablock terpolymers for targeted applications. While the glassy tBS blocks and rubbery HI blocks remain unchanged to maintain structural integrity, the middle block cation and anion chemistry were tailored to their desired applications. For instance, when employed as membranes for electroactive actuators^115–118^ or gas separation,^77,81^ the midblock contained covalently bonded sulfonate anions with paired protons or imidazolium cations.^82,83^ In contrast, when used as dry conductors in batteries,^61,68,70,84,119,120^ the midblock was modified to be hydrophobic and incorporated lithium, sodium, or imidazolium cations with paired (bis­(trifluoromethanesulfonyl)­imide) (TFSI) anions. Despite variations in cation and anion chemistries, in most cases, multi-ion conducting ABCBA pentablock terpolymers exhibited similar morphological behavior of microphase separation without long-range order, and the domain spacing increased with the addition of ILs and salts. Mobile ions preferentially incorporated into the ionic domains due to their stronger intermolecular interactions with the covalently bonded ionic moieties on the midblock. This was supported by the shift of the midblock glass transition temperature (*T*_g_) to lower temperatures, while the *T*_g_s of the end styrene blocks remained constant.^68,70,84,119^ It is worth noting that the addition of ILs influenced the phase behavior order in several studies.^68,77,84^ Dai et al.^77^ observed selective swelling in the midblock and improved nanostructure order with the impregnation of ILs in NEXAR. A transition from lamellar to cylindrical morphology was observed with increasing IL content (Figure 6a), similar to what has been observed in diblock copolymers swollen with selective solvents.^22,121^ Chen et al.^68^ investigated the morphology of Li salt/IL incorporated ABCBA pentablock terpolymers (chemical structure: Figure 4c) as solid polymer electrolytes. They reported an unusual decrease in domain spacing with increasing IL contents, while the morphology type remained unchanged, i.e., a poorly ordered body-centered cubic (BCC) spherical morphology with styrene (S)-ethylene propylene (EP) core–shell spheres embedded in a continuous ionic matrix. The decrease in domain spacing resulted from the formation of smaller S-EP spheres to accommodate the increase in IL volume without significantly stretching the midblock polymer chains. In both studies, the nonionic phase rearranged into nanostructures with higher interfacial areas, suggesting a lower enthalpy penalty from increasing interfacial areas between the ionic and nonionic domains compared to the entropy cost from the ionic midblock chain stretching. Additionally, Kim et al.^84^ observed a transition from microphase separation with no assigned morphology type to a lamellar morphology after the incorporation of Li salt/IL in a cross-linked ion-conducting ABCBA pentablock terpolymer (Figure 6b). Long-range ordering of lamellae was formed at a low degree of cross-linking (1% and 5%), attributed to the enhanced chain mobility with the presence of ILs, which promotes the rearrangement of molecular packing despite the presence of a cross-linked network.

**Figure 6:**
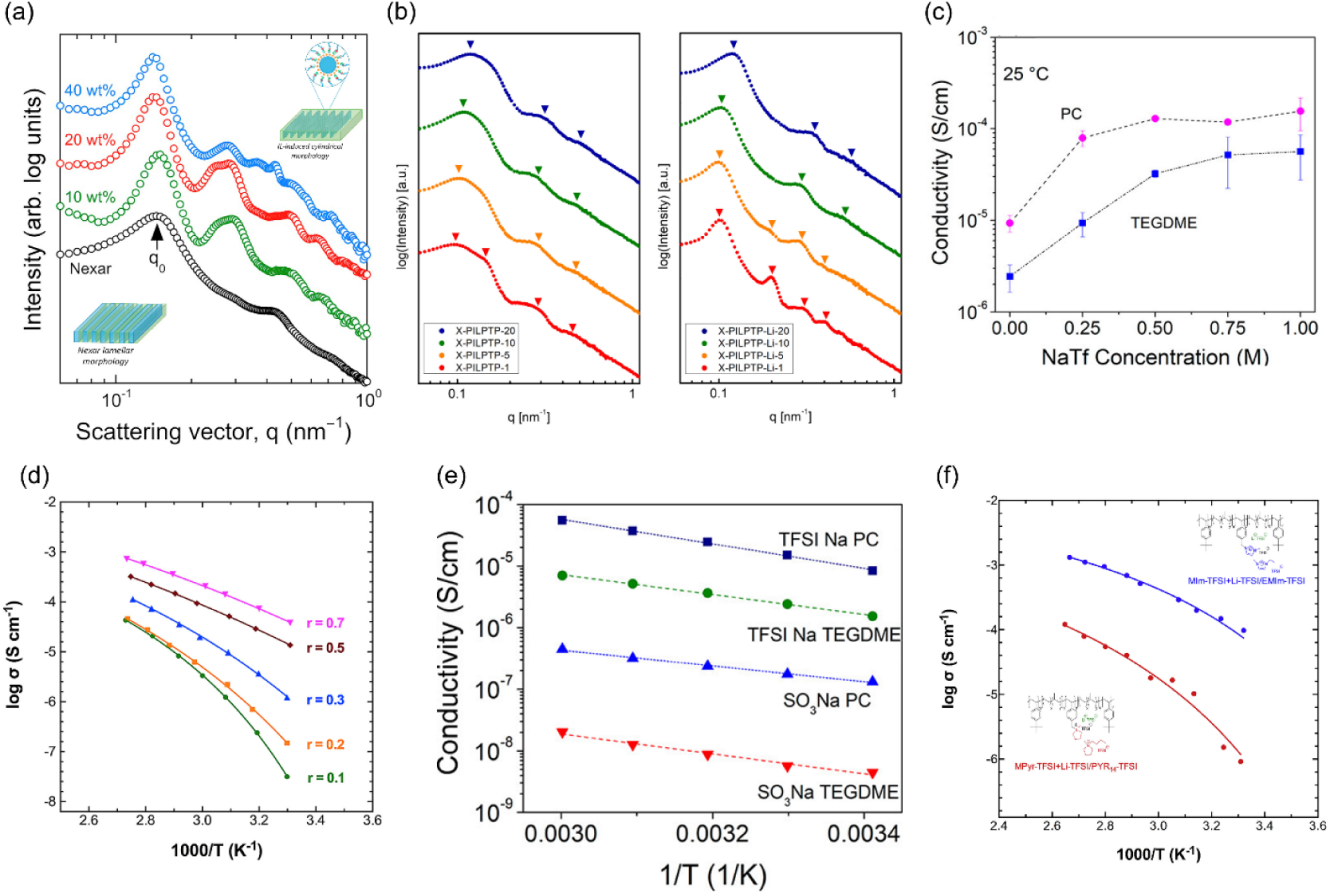
Examples of morphology and ion conductivity of multi-ion conducting ABCBA pentablock terpolymers. (a) Phase diagrams of IL-incorporated NEXAR at different IL contents. Adapted with permission from ref 77 (Copyright 2019 The Authors. Published by Elsevier B.V.). (b) SAXS profiles of cross-linked TFSI-conducting ABCBA pentablock terpolymers before and after Li salt/IL absorption. Reprinted with permission from ref 84 (Copyright 2025 The Author(s). Published by Wiley Periodicals LLC). (c) Ion conductivity of the sodium-conducting ABCBA pentablock terpolymer as a function of salt concentration. Adapted with permission from ref 61 (Copyright 2022 American Chemical Society). (d) Ion conductivity of the Li salt/IL incorporated ABCBA pentablock terpolymer as a function of IL concentration. Adapted with permission from ref 68 (Copyright 2020 Elsevier). (e) Ion conductivity of a sodium-conducting ABCBA pentablock terpolymer with different tethered anions. Reprinted with permission from ref 61 (Copyright 2022 American Chemical Society). (f) Ion conductivity of the Li salt/IL incorporated ABCBA pentablock terpolymer with different cations. Reprinted with permission from ref 119 (Copyright 2019 Elsevier).

While the addition of ILs influenced the ordering of nanostructures and ionic domain spacing, these subtle morphology differences generally did not significantly impact ion transport in multi-ion conducting ABCBA pentablock terpolymers. Rather, ion transport was more strongly influenced by the salt/IL concentration and the chemistry of the ionic moieties. Saito and coworkers^61^ examined the conductivity of a sodium-conducting ABCBA pentablock terpolymer plasticized by organic solvents as a function of salt concentration (chemical structure: Figure 4d). Ion conductivity increases with increasing salt concentration (Figure 6c), suggesting that ion conductivity is directly related to the number of mobile ions. Elabd and coworkers^68^ report that ion conductivity increases with increasing IL content and is less temperature-dependent at higher IL volume fractions (Figure 6d). At low IL compositions (i.e., molar ratio of IL to conductive block (*r*) ≤0.3), the temperature-dependent conductivity exhibited a VFT behavior similar to single-ion transport under dry conditions, indicating that ion mobility is governed by the polymer chain segmental motion. When the IL content was increased over a critical composition, the ionic conductivity exhibited Arrhenius behavior as a function of temperature, suggesting that the mobile ions move via a thermal ion hopping mechanism, similar to the water-assisted transport mechanism mentioned previously. This phenomenon was attributed to the combined effect of increased ion concentration and reduced *T*_g_ of the conducting phase (increase in the segmental motion of the polymer chains). Another factor that impacts the ion conductivity is cation and anion chemistries. Delocalized charges promote the dissociation of ions and subsequently increase the ion conductivity. For instance, ABCBA pentablock terpolymers tethered with the delocalized TFSI anions achieved over 2 orders of magnitude higher conductivity compared to those tethered with the sulfonate anions (Figure 6e).^61^ In another study, pentablock terpolymers incorporating ILs with unsaturated imidazolium cations achieved higher conductivity than those incorporating ILs with saturated pyrrolidinium cations (Figure 6f), attributed to delocalization of the positive charge on the imidazolium ring.^119^

### 2.3 Multi-Ion Conducting ABC Triblock Terpolymers

The next class of ion-conducting multiblock polymers, multi-ion conducting ABC triblock terpolymers, are salt- or IL-incorporated neutral ABC triblock terpolymers. Compared to commercially available ABCBA pentablock terpolymers, this class of polymers is often carefully designed and synthesized on a small scale, enabling the potential to form a diverse set of novel or unexpected nanostructures with long-range order attributed to (1) a wide selection of monomers and different block sequences with varying χ parameters, (2) a broad range of compositions across the phase diagram, and (3) a narrow dispersity achieved through precise synthesis. There are seven studies on multi-ion conducting ABC triblock terpolymers.^62–64,85,122–124^ A common observation across the studies is that they all contain a poly­(ethylene oxide) (PEO) block due to its compatibility with alkali metal salts and ability to facilitate ion transport.^58,62^

Epps et al.^62,85^ pioneered the systematic investigation of the morphological behavior of multi-ion conducting ABC triblock terpolymers (chemical structure: Figure 4e). Lithium perchlorate (LiClO_4_)-doped poly­(styrene-*b*-isoprene-*b*-ethylene oxide) (SIO) was compared to their neat triblock analogs to investigate the influence of salt on phase behavior and the order–disorder transition (ODT) temperatures (Figure 7a).^62^ The SIO chemistries and block sequence place these polymers in the category of frustrated systems (χ_AC_ < χ_AB_ < χ_BC_). Two variables in the study are the volume fraction of the PEO block (17 compositions, ϕ_PEO_ ranging from 0–33%) and the salt concentration ([O]/[Li] ranging from 48:1 to 1:1). At the same ϕ_PEO_, morphology was stable as a function of salt concentration up to [O]/[Li] = 3:1, while the ODT temperature and the size of the PEO domains largely increased with increasing salt concentration. At the same [O]/[Li], the phase diagram isopleth was significantly altered at varying PEO compositions. For example, at [O]/[Li] = 24:1, the loss of core–shell gyroid (CSG) and semiperforated lamellae (SPL) morphology was observed between PEO compositions of 0.207 and 0.280. The change in phase behavior was attributed to the increases in effective χ parameters and the coordination between PEO chains and lithium ions, resulting in stronger microphase separation. A further study explored the impact of block sequence on the phase behavior of doped SIO and ISO triblock terpolymers.^85^ Different from the SIO triblock terpolymers, neat ISO triblock terpolymers fall into the unfrustrated category (χ_AC_ > χ_AB_ > χ_BC_). Only three morphology types (LAM_2_, noncubic network, and LAM_3_) of typical diblock copolymers and their three-phase analogues were observed, attributed to the change in the interfacial tension between blocks caused by the different block sequence. Upon the addition of salt, the polarity of the PEO block significantly increases, leading to significantly increased χ_SO_ and χ_IO_, while χ_SI_ remained constant (unfrustrated category, χ_AC_ > χ_BC_ > χ_AB_). The overall PEO interfacial area decreases, driving additional chain stretching and leading to a morphological transition from a noncubic network to a CSC structure. However, despite a thorough investigation of the phase behavior, these studies did not include conductivity measurements to investigate the relationship between morphology and ion transport in these polymers.

**Figure 7:**
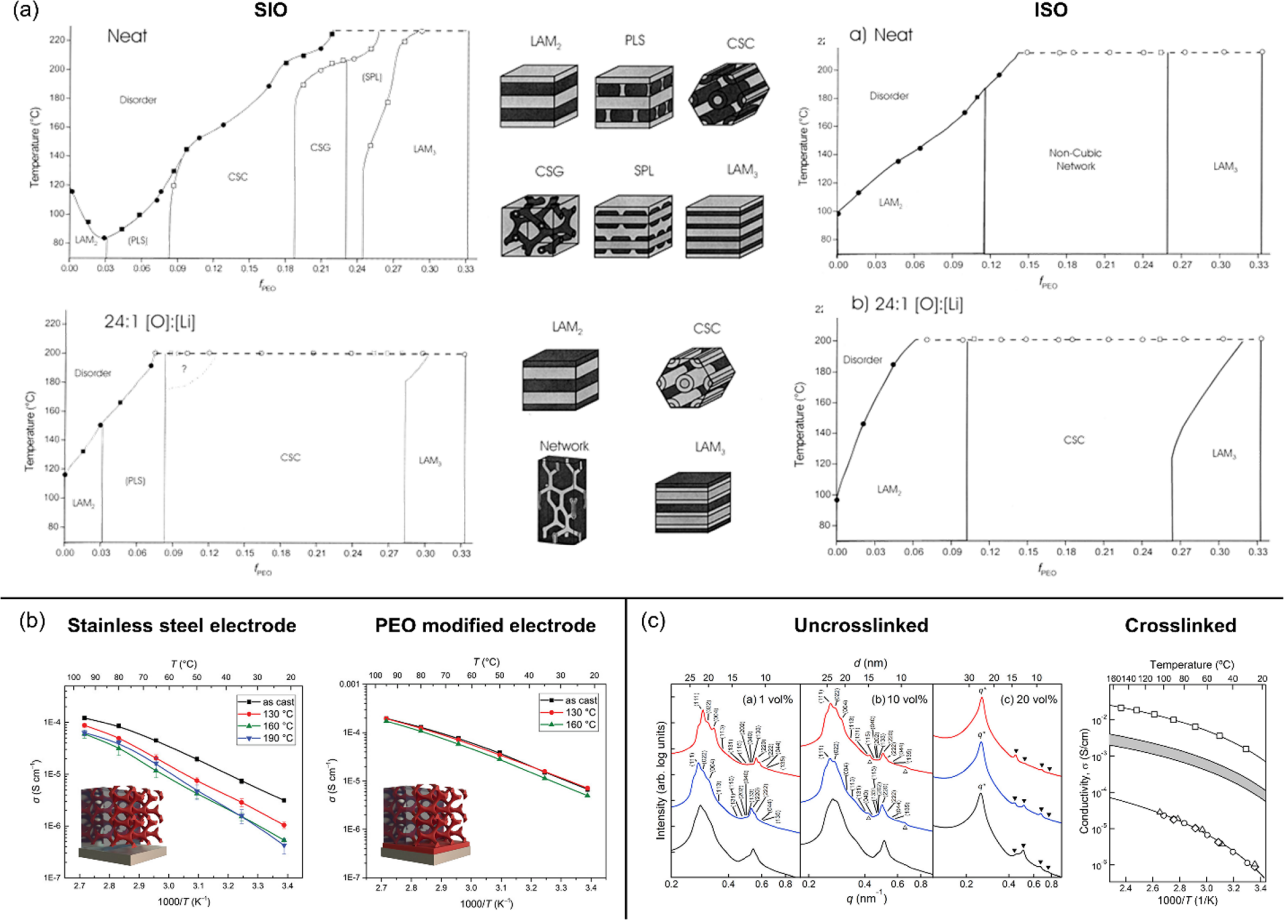
Examples of morphology and ion conductivity of multi-ion conducting ABC triblock terpolymers. (a) Phase diagrams of neat and LiClO_4_-doped SIO and ISO triblock terpolymers. Adapted with permission from ref 62 (Copyright 2002 American Chemical Society) and ref 85 (Copyright 2003 American Chemical Society). (b) Ion conductivity of LiTFSI-doped ISO measured using stainless steel electrodes and PEO-modified electrodes. Adapted with permission from ref 63 (Copyright 2019 WILEY-VCH Verlag GmbH & Co. KGaA, Weinheim). (c) SAXS profiles of IL-doped un-cross-linked INSO membrane and ion conductivity of IL-doped cross-linked INSO membrane. Note the ion conductivity of IL-doped un-cross-linked INSO membrane was not measurable. Adapted with permission from ref 64 (Copyright 2014 American Chemical Society).

Sutton et al.^63^ investigated how the morphology, specifically at the polymer electrolyte and blocking electrode interface, affects the conductivity of a multi-ion conducting ABC triblock terpolymer. In this study, the LiTFSI-doped ISO polymer (chemical structure: Figure 4f) exhibited an isotropic network morphology consisting of a PS matrix with alternating gyroids of PI and PEO. Conductivity of polymer films measured with standard stainless-steel blocking electrodes significantly decreased upon thermal annealing above the *T*_g_ of PS, while conductivity measured with PEO-modified electrodes remained stable across the temperature range. The authors hypothesized that the decrease in conductivity was attributed to a morphological change induced by the surface preference of PS on the electrode surfaces, while the morphology of the bulk polymer film remained unchanged. A thin layer of PS on the nanometer scale may form on the steel electrodes and block the contact between the ion-conducting PEO phase and the electrodes (Figure 7b). PEO-modified electrodes promote the surface restructuring of salt-doped ISO, enabling a continuous pathway for ion transport between the electrode and the bulk electrolyte membrane.

Using ILs instead of lithium salts as dopants, IL-incorporated ABC triblock terpolymers target a synergistic interaction between glassy polymers and conductive ILs to achieve ion gels exhibiting both high mechanical strength and high ionic conductivity.^64,122,123^ While two other studies focused on the impact of ILs on the mechanical properties of ABC triblock terpolymers consisting one or more thermoresponsive blocks, McIntosh et al.^64^ investigated the morphology, modulus, and conductivity of an IL/ABC triblock terpolymer system consisting of poly­(isoprene-*b*-(styrene-*co*-norbornenylethylstyrene)-*b*-ethylene oxide) (INSO) and 1-ethyl-3-methylimidazolium bis (trifluoromethylsulfonyl)­imide (EMITFSI) (chemical structure: Figure 4g). A morphology transition from highly ordered O^70^ (*Fddd*) (neat INSO polymer) to hexagonally packed cylinders (INSO/IL blends) (Figure 7c) was observed, especially at higher IL concentrations, attributed to the interfacial energy change between blocks with increasing IL content. However, high ionic conductivity was not observed in spite of high ion concentration with the addition of ILs in the polymers, possibly due to preferential wetting of the stainless-steel electrodes by the I or N-*co*-S block, or the loss of highly ordered O^70^ (*Fddd*) morphology that results in isolated or nonpercolating conducting domains.

### 2.4 Single-Ion Conducting ABC Triblock Terpolymers

To date, there are only four reports on single-ion conducting ABC triblock terpolymers, where three are lithium or TFSI conducting polymers for battery applications,^35,65,66^ and one is hydroxide-conducting AEMs for fuel cell applications.^67^ Mayes and coworkers^65^ first synthesized a single-ion conducting ABC triblock terpolymer, poly­(lauryl methacrylate (LMA)-*b*-lithium methacrylate (LiMA)-*b*-(oxyethylene)_9_ methacrylate (OEM)) (chemical structure: Figure 4h), in 2005. The polymer was compared to two analogous diblock polymers (P­(LMA-*r*-LiMA)-*b*-POEM and PLMA-*b*-P­(LiMA-*r*-OEM)) to study the influence of ion placement on the morphology and conductivity. Despite the absence of a well-defined morphology in all three polymers, the triblock terpolymer exhibited stronger phase separation compared to the diblock copolymers (Figure 8a). PLMA-*b*-PLiMA-*b*-POEM and P­(LMA-*r*-LiMA)-*b*-POEM showed 1 order of magnitude higher conductivity than PLMA-*b*-P­(LiMA-*r*-OEM), attributed to phase separation between the POEM and PLiMA blocks. The carboxylate anions covalently bonded to the PLiMA block were separated from the POEM block, promoting the dissociation of lithium ions from carboxylate anions and their transport within the ether-rich POEM domains.

**Figure 8:**
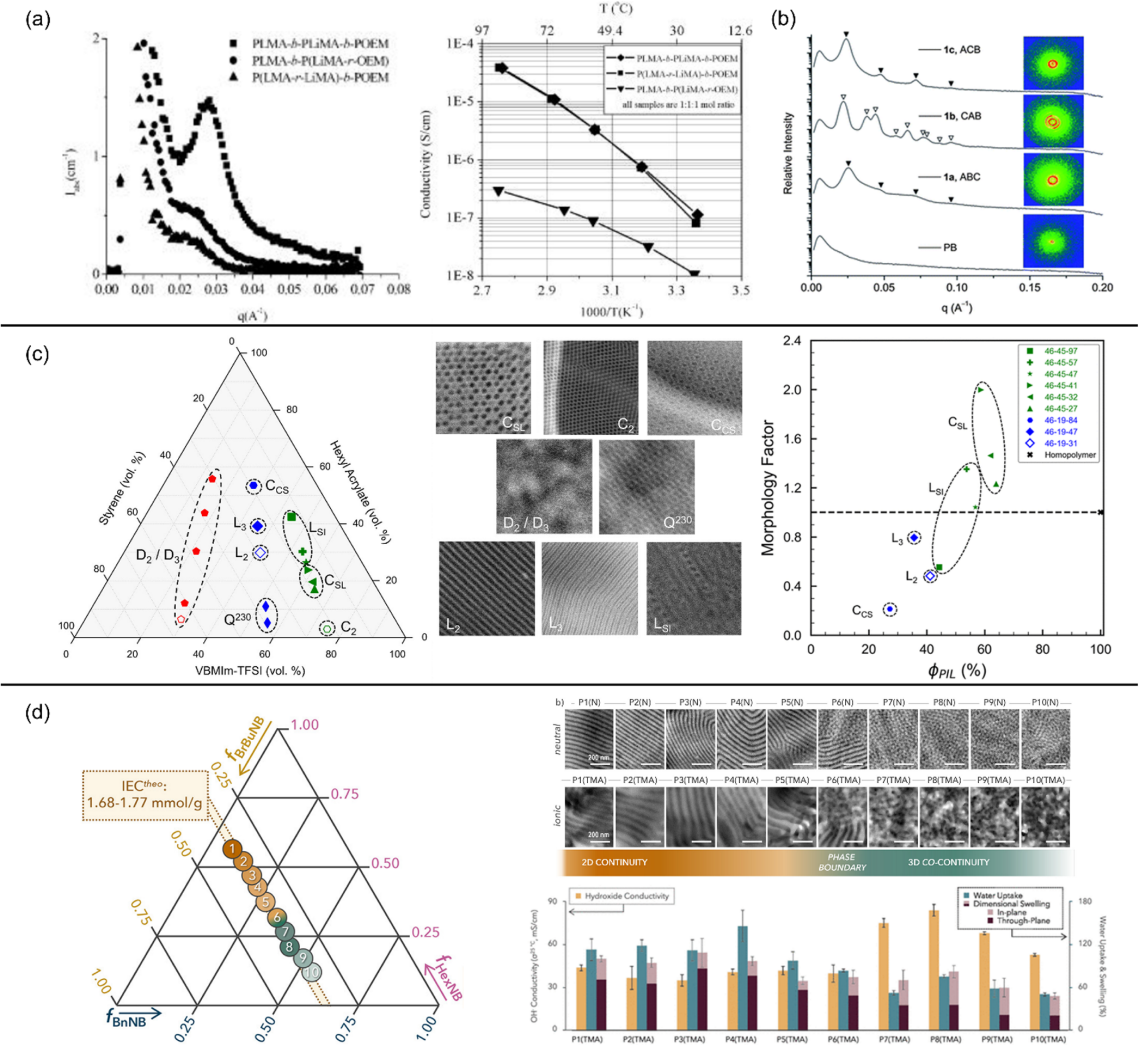
Examples of the morphology and ion conductivity of single-ion conducting ABC triblock terpolymers. (a) SAXS profiles and ion conductivity of PLMA-*b*-PLiMA-*b*-POEM and its analogous diblock copolymers. Adapted with permission from ref 65 (Copyright 2005 IOP Publishing). (b) SAXS profiles of a single-ion conducting ABC triblock terpolymer with different block sequences (ABC vs CAB vs ACB). Adapted with permission from ref 66 (Copyright 2013 The Royal Society of Chemistry). (c) Morphology phase diagram, TEM images, and morphology factor (*f*) of poly­(S-*b*-VBMImTFSI-*b*-HA). Adapted with permission from ref 35 (Copyright 2024 The Authors. Published by American Chemical Society). (d) Phase diagram, TEM images, and ion conductivity of a single-ion conducting polynorbornene-based ABC triblock terpolymer. Adapted with permission from ref 67 (Copyright 2025 American Chemical Society).

In 2013, Gin and coworkers^66^ developed the first set of single-ion conducting ABC triblock terpolymers with ordered nanophase morphology and investigated the impact of block sequence (ABC vs CAB vs ACB) on the melt-state morphology. The polymers contain a norbornene-based backbone with a neutral hydrophobic block with a long alkyl chain (A), an ionic block with imidazolium cation and TFSI anion (B), and a neutral hydrophilic block containing polyethylene glycol (C) at a molar block composition of 1:1:1 (chemical structure: Figure 4i). ABC and ACB exhibited lamellar morphology, while CAB showed a hexagonally packed cylindrical morphology, both with long-range order (Figure 8b). This observation corroborates the frustration theory in neutral ABC triblock terpolymers. Specifically, in this study, χ_AB_ and χ_AC_ are larger than χ_BC_, indicating that ABC and ACB are in the unfrustrated category and CAB is in the frustrated category. The enthalpic preference of the BC interfaces contradicts the separation of B and C in block sequence and results in different chain packing and nanostructures in CAB. However, an equimolar composition of the three blocks only results in morphologies similar to those observed in diblock copolymers. The ionic conductivity of the polymers was not measured and the morphology-conductivity relationship was not explored in this study.

To provide a deeper understanding of the morphology–conductivity relationship of single-ion conducting ABC triblock terpolymers, our group recently constructed a ternary phase diagram containing 17 compositions of a single-ion conducting ABC triblock terpolymer and measured the temperature-dependent conductivity of the polymers.^35^ The block chemistries were carefully selected, where styrene (S) was the mechanically robust A block, vinylbenzyl methylimidazolium bis (trifluoromethanesulfonyl)­imide (VBMImTFSI) was the ion-conductive B block, and hexyl acrylate (HA) was the flexible C block (chemical structure: Figure 4j). These polymers are in the highly frustrated category with this combination of chemistries containing the immiscible ionic block in the middle (χ_BC_ ≥ χ_AB_ ≥ χ_AC_), resulting in a diverse library of ordered morphologies at different block compositions. Among the nine morphology types observed in this study (Figure 8c), there are typical AB diblock morphologies and their direct three-domain analogues, such as two-phase hexagonally packed cylinders (C_2_), two-phase lamellae (L_2_), three-phase lamellae (L_3_), and a three-phase hexagonal superlattice of cylinders (C_SL_). In addition, morphologies that are not observed in AB diblocks or nonfrustrated ABC triblocks were also observed, including core–shell hexagonally packed cylinders (C_CS_), core–shell double gyroid (Q^230^), and spheres-in-lamellae (L_SI_), confirming the highly frustrated nature of these polymers. Similar glass transition behavior was observed in polymers with well-defined phase separation, suggesting that differences in conductivity arise primarily from morphology and the volume fraction of the conducting phase rather than from differences in chain segmental mobility. Morphology factor (*f*) changed significantly with only small differences in the conducting volume fraction, indicating that the morphology types have a substantial influence on ion transport. Particularly, C_SL_ morphology achieved an exceptionally high morphology factor of *f* = 2.0, which can be attributed to the formation of 3D continuous ion-conducting nanochannels of ca. 6–10 nm. These well-connected narrow nanochannel ionic pathways increased the local ion concentration and thereby resulted in accelerated ion transport. A similar confinement effect has also been observed in block copolymer electrolytes containing two block chemistries.^125–127^

More recently, Coates and coworkers^67^ developed a series of high molecular weight polynorbornene ABC triblock terpolymer AEMs containing an A block functionalized with alkyl groups, a B block functionalized with benzyl groups, and a C block functionalized with bromobutyl groups, which were later functionalized with trimethylamine to form the conducting block (chemical structure: Figure 4k). Ten block compositions were achieved by adjusting the block ratio between A and B while maintaining the C block composition constant at 0.35 (Figure 8d). Similar molecular weights and IECs were achieved to explore the morphology impact exclusively. Locally ordered 2D-continuous lamellar and 3D-continuous network morphologies without exceptionally long-range orders were observed in the neutral triblock terpolymers, with the phase transition occurring at a block composition of *f*_A_ = 0.35 and *f*_B_ = 0.30. Interestingly, a decrease in the degree of ordering was observed for the lamellar morphology after functionalization, while the order of the network phase morphology was better preserved, as shown in the SAXS profiles. Morphology significantly influenced the ion conductivity, water uptake, and dimensional swelling. AEMs with 3D-continuous network morphology achieved almost twice the conductivity and half of the water uptake and dimensional swelling of the AEMs with 2D-continuous lamellar morphology. This difference was attributed to more efficient ionic transport and enhanced structural support through both 3D-continuous ionic and hydrophobic domains. This study further confirms the role of 3D-continuous structures in accelerating ion conduction and reveals the advantages of ABC triblock terpolymers to achieve morphologies with well-defined domain continuity.

### 2.5 Discussion

#### 2.5.1 Multi-Ion Conducting vs Single-Ion Conducting Multiblock Terpolymers

In Sections 2.1–2.4, studies on ion-conducting multiblock terpolymers categorized by the number of mobile ions in the polymers (single-ion and multi-ion) and their chain architecture (ABCBA and ABC) were reviewed. When comparing multi-ion conducting and single-ion conducting multiblock terpolymers, the incorporation of ILs and salts significantly increases the mobile ion concentration and alters the intermolecular interactions (e.g., mobile ion–mobile ion, mobile ion–tethered ion, mobile ion–polymer chain), resulting in (1) higher ion conductivity compared to their analogous single-ion conducting multiblock terpolymer, (2) a shift in equilibrium phase behavior with a limited set of conventional morphology types, and (3) less morphological impact on ion transport due to the lack of well-defined network morphology with continuous ionic domains. High ion conductivity results from the motion of both cations and anions, creating complex ion diffusion and clustering behavior in multi-ion conducting multiblock terpolymers. This phenomenon is specifically of interest in battery applications. Chen et al.^68^ measured the self-diffusion of mobile ions (*D*) in IL-doped ABCBA pentablock terpolymer electrolytes as a function of IL concentration (*r* = [IL]/[conducting block] (mol/mol)). The diffusion of lithium ions in polymer electrolytes at high IL concentration (*r* ≥ 0.5) was half of that at low IL concentration (*r* = 0.2), while the diffusion of the TFSI anion is higher at *r* ≥ 0.5, resulting in a lower lithium ion transport number (i.e., the fraction of current transported by lithium ions). The mobility of lithium ions decreases with increasing IL concentration, and the anions start to accumulate at the electrode surface. The formation of concentration gradients leads to a significant increase in cell overvoltage and a decrease in electrochemical stability of the multi-ion conducting multiblock terpolymers. Additionally, anion-rich ion clusters containing lithium ions, i.e., (LiX_m+1_)^m–^, may form and move lithium ions in the opposite direction, resulting in reduced or even negative lithium transference numbers. While there is no report on lithium transference numbers in multi-ion conducting multiblock terpolymers to date, significantly reduced battery performance has been observed in other multi-ion conducting polymer systems with low or negative lithium transference numbers.^128–130^

In contrast, single-ion conducting multiblock terpolymers contain only one type of mobile ion, and the ion transport number and transference number are both close to unity. They can suppress lithium dendrite growth by mitigating the formation of concentration gradients and promoting uniform Li deposition, achieving enhanced battery performance with longer lifetimes.^131–133^ Theoretical calculations also suggest that the ion conductivity requirement for single-ion conducting polymer electrolytes (transference number = 1) is one-tenth of the requirement for multi-ion conducting polymers (transference number = 0.2).^134^ However, challenges remain in developing single-ion conducting polymers with high room-temperature ion conductivity due to their fundamental limitations of (1) slow segmental motions and high *T*_g_s, (2) low dielectric constant with high electrostatic interactions, and (3) limited mobile ion concentration, as suggested in a prior perspective by Bocharova and Sokolov.^135^ Developing single-ion conducting multiblock terpolymers with long-range continuous 3D nanoscale ionic channels and very high local ion concentrations is one of the most promising approaches to achieve this goal.

#### 2.5.2 Ion-Conducting ABC vs ABCBA Multiblock Terpolymers

While the number of studies on ion-conducting ABCBA pentablock terpolymers outnumbers ABC triblock terpolymers, the studies are almost exclusively focused on the commercially available NEXAR polymers and polymers modified from the same neutral precursors. The polymers were constrained to symmetric pentablock terpolymers with fixed backbone chemistry, molecular weight, and block composition. In addition, sulfonation or postpolymerization functionalization results in a random distribution of ionic groups along the midblock polymer chains. Therefore, the equilibrium morphologies of ion-conducting ABCBA pentablock terpolymers in the existing studies are narrowed to disordered microphase separation, spheres, or lamellae. Only a limited set of parameters, such as IECs, processing methods, and ionic group chemistry, can be adjusted to tune the nanostructure and continuity of the ionic domains. Future studies with careful design and precise synthesis are needed to realize the morphological potential of ion-conducting ABCBA pentablock terpolymers.

In contrast, the few reports on ion-conducting ABC triblock terpolymers have showcased a variety of morphology types, including some unexpected types, which have a distinct effect on ion transport. As mentioned previously, the morphology factor reveals the morphology–conductivity relationship by normalizing the conductivity of the material with the intrinsic conductivity and the volume fraction of the conducting phase.^15,136–139^ While several ion-conducting diblock copolymer with network morphology have reported relatively high morphology factors (0.5 < *f* < 1),^125–127^ a series of single-ion conducting ABC triblock terpolymers were able to achieve unusually high morphology factors above 1.0, owing to the formation of 3D continuous nanochannel pathways that provide high local ion concentrations. This highlights the potential of ion-conducting ABC triblock terpolymers to accelerate ion conduction through the diverse morphologies that they can achieve.

## 3 Future Outlook

To answer the question at the beginning: How does the incorporation of charge affect the morphology and subsequently the ion conduction of multiblock terpolymers with three distinct chemistries? Observations from Section 2 indicate that a small shift in block compositions and frustration leads to a significant change in self-assembly behavior and ion conductivity. The unusually high morphology factors achieved by polymers with hexagonal superlattice and spheres-in-lamellae morphologies suggest the potential of ion-conducting multiblock terpolymers to form a wide range of 3D continuous network morphologies for fast ion transport with their diverse set of molecular parameters. In addition, it is also worth noting that the spheres-in-lamellae morphology was not previously observed in experimental studies, but only recently discovered in a highly frustrated ion-conducting ABC triblock terpolymer. However, while most morphologies in Figure 2 have been observed in neutral multiblock terpolymers, to date, only 12 morphologies have been observed in ion-conducting multiblock terpolymers, due to the limited number of studies with only a small set of chemistries has been explored.

Many questions remain. How many additional new morphologies can be observed in ion-conducting multiblock terpolymers? How many of these morphologies contain 3D continuous narrow ionic channels? What is the impact of these morphologies on ion conductivity and, subsequently, electrochemical performance? Therefore, constructing a comprehensive phase diagram across a broad range of chemistries and compositions will provide deeper insights into these questions.

However, to leverage the full potential of ion-conducting multiblock terpolymers, the significant number of molecular variables in polymer design is challenging. Characteristics, such as different block sequences, high molecular weight, asymmetry in block length, and controlled ion distributions, rely on advanced synthetic approaches to achieve precise synthesis with narrow dispersity. While polymerization techniques have evolved significantly over the past decades, challenges remain due to the complexity of ion-conducting multiblock terpolymer systems with different monomer compatibility and solubility, meticulously designed chain architecture and block length, and the significant quantity of synthesis and characterization required to achieve a wide range of block compositions to construct the phase diagram. Theoretical and computational studies are powerful tools to explore the morphological behavior and guide the selection of targeted chemistry and compositions for experimental synthesis while significantly reducing experimental time. Targeted synthesis guided by computational design can discover an unexplored, diverse set of morphologies in ion-conducting multiblock terpolymers with three distinct chemistries and answer many unanswered questions, possibly unlocking a new set of materials with ultrahigh ion conductivities and electrochemical performance.
